# Local radio to promote mental health awareness: a public health initiative

**DOI:** 10.1192/bjo.2019.51

**Published:** 2019-07-12

**Authors:** Karen A. Cocksedge, Joshana Guliani, William Henley, Tamsyn Anderson, Sara Roberts, Laurence Reed, Daphne Skinnard, Sarah Fisher, Beth Chapman, Joanna Willcox, Ellen Wilkinson, Richard Laugharne, Rohit Shankar

**Affiliations:** Speciality Trainee in General Adult Psychiatry, Cornwall Partnership NHS Foundation Trust; and Livewell Southwest, Mount Gould Local Care Centre, UK; Core Trainee in Psychiatry, Royal Cornwall Hospital; and South London and Maudsley NHS Foundation Trust, Bethlem Royal Hospital, UK; Professor in Medical Statistics, Exeter Medical School, Knowledge Spa, Royal Cornwall Hospital, UK; Director of Primary Care, Cornwall Partnership NHS Foundation Trust, UK; Public Health Lead for Mental Health, Public Health Cornwall, UK; Broadcaster, BBC Cornwall, UK; Assistant Managing Editor, BBC Cornwall, UK; Communications Director, Cornwall Partnership NHS Foundation Trust, UK; Consultant Psychiatrist, Cornwall Partnership NHS Foundation Trust, UK; Community Nurse in Forensic Psychiatry, Cornwall Partnership NHS Foundation Trust, UK; Medical Director, Cornwall Partnership NHS Foundation Trust, UK; Consultant Psychiatrist, Cornwall Partnership NHS Foundation Trust; and Honorary Senior Clinical Lecturer, Exeter Medical School, Knowledge Spa, Royal Cornwall Hospital, UK; Consultant Neuropsychiatrist, Cornwall Partnership NHS Foundation Trust; and Senior Clinical Lecturer, Exeter Medical School, Knowledge Spa, Royal Cornwall Hospital, UK

**Keywords:** Mental disorders, mental illness stigma, public mental health, public opinion, mass media

## Abstract

**Background:**

Public health strategies have focused largely on physical health. However, there is increasing recognition that raising mental health awareness and tackling stigma is crucial to reduce disease burden. National campaigns have had some success but tackling issues locally is particularly important.

**Aims:**

To assess the public's awareness and perception of the monthly BBC Cornwall mental health phone-in programmes that have run for 8.5 years in Cornwall, UK (population 530 000).

**Method:**

A consultation, review and feedback process involving a multiagency forum of mental and public health professionals, people with lived experience and local National Health Service trust's media team was used to develop a brief questionnaire. This was offered to all attendees at two local pharmacies covering populations of 27 000 over a 2-week period.

**Results:**

In total, 14% (95% CI 11.9–16.5) were aware of the radio show, 11% (95% CI 9.0–13.1) have listened and the majority (76%) of those who listened did so more than once. The estimated reach is 70 000 people in the local population, of whom approximately 60 000 listen regularly. The show is highly valued among respondents with modal and median scores of 4 out of 5.

**Conclusions:**

Local radio is a successful, cost-effective and impactful way to reach a significant proportion of the population and likely to raise awareness, reduce stigma and be well received. The format has been adopted in other regions thus demonstrating easy transferability. It could form an essential part of a public health strategy to improve a population's mental well-being.

**Declaration of interest:**

W.H. received support from the National Institute for Health Research (NIHR) Collaboration for Leadership in Applied Health Research and Care (CLAHRC) for the South West Peninsula UK. The views expressed in this publication are those of the authors and not necessarily those of the NHS, the NIHR or the Department of Health. L.R. and D.S. were involved in delivering the programmes but had no role in their evaluation.

## National perspective

Historically, UK public health strategies have largely focused on physical health, but in 2010, the Royal College of Psychiatrists issued a Parliamentary briefing that highlighted the importance of also viewing mental health as a core public health issue.^1^ Mental health is the single largest source of burden of disease in the UK^1^ and as such promotion of good mental health and prevention of mental ill health is crucial. Stigma and discrimination against people with mental health difficulties remains a significant concern but public education has been shown to be helpful in tackling this.^2^

Mass media campaigns are an important strategy for public education^3^ and have been shown to have an influential role in shaping public views.^4^ Such campaigns have focused largely on delivering messages regarding physical health, such as stop smoking campaigns.^5^ However, the Time to Change programme was launched in England in 2009 and aimed to reduce stigma and discrimination of mental health through a mass media campaign and local contact events.^6^ Outcomes from this were mixed but particular success was noted from local events, with the suggestion that local and national campaigns might work synergistically to improve outcomes.^6^ Engagement of the public locally through local radio carries significant potential since a high proportion of the population listen to local radio.^7^

## Cornwall perspective

In the county of Cornwall in the UK, it is thought that over half of the total adult population listen to local BBC radio station, Radio Cornwall, though the numbers fluctuate week on week and year on year.^8^ Cornwall is a rural County that is traditionally divided into West, Mid and East Cornwall. It has a population of approximately 530 000 and only 1.8% of the population are from Black and ethnic minority groups.^9^ About 15% of Cornwall is among the highest fifth of deprivation in England and has the second highest suicide rate of all 326 local authorities in England.^10,11^

Since 2011, a joint initiative has been running between BBC Radio Cornwall and the local National Health Service (NHS) mental health trust to present a monthly phone-in programme on various mental health topics that attempts to raise awareness.^12,13^ There are no formal contractual arrangements with the local NHS trust; instead this is a coproduction between the BBC and the NHS of a voluntary nature. The format of the programmes has been previously described^13^ but in summary, they form part of the daily BBC Cornwall lunchtime show, which is usually hosted by presenter L.R., and are aired between 13.00 h and 14.00 h on the first Wednesday of every month.

The programme starts with a 5 min pre-recorded patient experience, followed by two NHS professionals as guests being asked questions by the BBC presenter and any callers that phone into the programme. The callers typically share their own experiences in addition to asking questions, which adds to useful and interesting discussion. The NHS professionals are typically a consultant psychiatrist and a non-medical clinician (such as a nurse or psychologist). Each programme covers a different topic of mental health and some of the more common programmes that have been covered and later repeated have include the following topics: depression, anxiety, post-traumatic stress disorder, obsessive–compulsive disorder, psychosis, bipolar affective disorder, dementia, personality disorders, self-harm and suicide, eating disorders, children's mental health, perinatal psychiatry, intellectual disability, alcohol and drug problems, and mental and physical health.

A schedule of programmes is organised in advance (currently by J.W., previously by K.A.C. and then previously B.C.) and the appropriate NHS professionals as guests, and willing patients for the pre-recorded interview are recruited accordingly. The majority of topics are generalisable to a wide audience (at a national or even international setting) but we have also included some topics that relate specifically to local services; however, even in the latter cases, these could be adapted to reflect equivalent services in an alternative location.

As a result of continued interest from the BBC, the programmes have run consistently since 2011 and in 2019 celebrated the 100th show. A survey of callers to the programme demonstrated that the vast majority found the show beneficial in delivering public education.^13^ However, attempts made to date by the BBC to survey the opinion of listeners have failed because of poor response rates.

This study has been co-designed and co-produced between the NHS and the BBC (involving presenter, L.R. and BBC Radio Cornwall's assistant managing editor, D.S.). Its aim was to assess the public's awareness and perception of the monthly BBC Cornwall mental health phone-in programmes. From those sampled, the primary objective was to determine the percentage of people who have listened to the show at least once since the programmes began and the value that listeners place on the show. A secondary objective was to assess the demographics of listeners compared with non-listeners in the sampled population including their mental health status. To the best of our knowledge, there are no other comparable data to assess public awareness and perception on any other radio mental health programmes.

## Method

A consultation, review and feedback process involving a multiagency forum of mental health professionals, public health professionals, people with lived experience and the local NHS trust's media team was used to develop a brief questionnaire. The final format of the questionnaire is shown in Appendix 1.

It is thought that the reach of BBC Radio Cornwall is approximately 300 000 people, of whom at least one-third listen to the BBC lunch time programme during which the monthly mental health phone-in is aired.^8^ This is an estimated prevalence of 20% of Cornwall's adult population. A sample size of 247 would be required to estimate this prevalence with a 5% error margin and a 95% confidence interval. Further calculations show that a sample size of 195 in two equally sized demographic groups would be required to detect a reduction in prevalence from 20% to 10% across the two groups with 80% power at a 5% significance level. Hence, it was felt that at least 400 completed questionnaires would allow a sufficiently precise estimation of the proportion of the Cornish population who listen to the mental health phone-in as well as making it possible to compare proportions across common demographic characteristics. The sample population was chosen from general practice (GP) patients attending their local pharmacy, such that pharmacy staff could suggest questionnaire completion while patients waited to collect a prescription. With an estimated responder rate of approximately 20%, 2000 people would need to be approached to achieve the required sample size. It is important to note, however, that in choosing pharmacy attenders as our sample population, this potentially skews our data towards those with health-related difficulties or at least those engaged in health-seeking behaviours when compared with the general population of Cornwall. This is considered further within discussion of the limitations of this study.

Two GP surgeries with attached pharmacies were chosen: one in West Cornwall (Hayle) with a practice registered population of approximately 11 000 patients and one in Mid Cornwall (Newquay) with a practice registered population of approximately 16 000 patients. Both pharmacies offered the questionnaire for a 2-week period during regular working hours, a time period that was estimated from average daily pharmacy attendances to allow at least 2000 people to be approached. From the prescriptions dispensed, the pharmacies were later able to estimate the actual number of people who attended during the study period to identify the completion rate for the questionnaires.

For comparison of categorical variables, a χ^2^-test was performed in all cases except where the expected value for the number of respondents in any cell is less than five respondents, in which case a Fisher's exact test was used. A Student's *t*-test was performed to compare the means of continuous variables. Throughout this paper, *P* < 0.05 is assumed to represent statistical significance. As this is an exploratory study, no formal adjustments (for example Bonferroni correction) were made for multiple testing.

### Ethics and participant consent

No ethical permission was required as this was a survey to feedback on an ongoing public programme. The project was registered as a service evaluation at the local NHS trust. All participants were advised that participation was voluntary and that their replies were anonymous. Consent was implicit by the return of the survey form. Anonymity was assured by having a drop box at each pharmacy and no names or addresses were collected. We also used the NHS health research authority tool (http://www.hra-decisiontools.org.uk/research/index.html), which also helped confirm that no ethical permission was needed for this project

## Results

From the prescriptions dispensed in Hayle, 590 patients attended the pharmacy during the study period and of these, 265 questionnaires were completed (45%). In Newquay, 2800 patients attended the pharmacy and of these, 629 questionnaires were completed (22%). Overall, 894 completed questionnaires (26%) were obtained from a total of 3390 patients.

The demographics of the respondents are reported in Table 1. No statistically significant difference was found between respondents of the two practices with regards to baseline characteristics of age, gender and employment status. Of the entire sample, 31% of respondents consider that they have been affected by mental health difficulties, 40% know a friend or relative who has been affected by mental health difficulties and 37% of respondents consider that neither they nor their friends nor family have ever been affected by mental ill health. *Post hoc* analysis comparing rates of mental illness in three age groups (18–35; 36–55; ≥56 years) showed that the 18- to 35-year-old respondent group consider that they have a significantly higher rate of mental illness than the ≥56 years respondent group (43% *v.* 15%; *P* < 0.00001). The Newquay respondents reported significantly higher rates of mental illness than the Hayle respondents (33% *v.* 26%; *P* = 0.040).

**Table 1:** Demographics of the sample

Demographic	All respondents (%) (*n* = 894)	Hayle (%) (*n* = 265)	Newquay (%) (*n* = 629)	Difference between Hayle and Newquay populations, *P*
Age, *n* (%)				
All^a^	891	265	626	
18–25 years	63 (7.1)	24 (9.1)	39 (6.2)	
26–35 years	150 (17)	38 (14)	112 (18)	
36–45 years	143 (16)	41 (15)	102 (16)	
46–55 years	165 (19)	39 (15)	126 (20)	
56–65 years	164 (18)	62 (23)	102 (16)	
>65 years	206 (23)	61 (23)	145 (23)	
Best estimate of age:^b^ years, mean (s.d.)	50.6 (16.8)	51.1 (17.1)	55.7 (16.7)	0.57
Gender, *n* (%)				
All^a^	892	263	629	
Men	331 (37)	104 (40)	227 (36)	
Women	558 (63)	157 (60)	401 (64)	
Other	3 (0.34)	2 (0.76)	1 (0.16)	
Gender distribution				0.30
Employment status, ^c^ *n* (%)				
All^a^	890	262	628	
Employed	444 (50)	129 (49)	315 (50)	
Unemployed	78 (8.8)	24 (9.2)	54 (8.6)	
Stay at home parent/carer	80 (9.0)	25 (9.5)	55 (8.8)	
Student	32 (3.6)	14 (5.3)	18 (2.9)	
Retired	267 (30)	74 (28)	193 (31)	
Employment distribution				0.55
Mental health status, *n* (%)				
All^a^	891	264	627	
Affected by mental health difficulties – self	273 (31)	68 (26)	205 (33)	
Friend/relative affected by mental health difficulties	358 (40)	98 (37)	260 (41)	
Both self and friend/relative affected by mental health difficulties	73 (8.2)	17 (6.4)	56 (8.9)	
Neither self nor friend/relative affected by mental health difficulties	333 (37)	115 (44)	218 (35)	
Mental health distribution				
Comparison of those with mental health difficulties in self/other versus those where neither self/other affected	–	–	–	0.013*
Comparison of those with mental health difficulties in self versus those with no mental health difficulties in self	–	–	–	0.040*
Comparison of those with a friend/family with mental health difficulties versus those with no mental health difficulties in friend/family	–	–	–	0.23

a. Data is missing for some respondents as they did not answer all questions.

b. The best estimate of the mean age assumes that the ages are evenly distributed in each class. For the >65 years age group, the class is assumed to be the age range 66–81 years, which uses the mean age of death based on data from the Office of National Statistics.^11^

c. Some respondents ticked more than one type of employment status, hence the totals are greater than 100%.

*P < 0.05.

The results from the questionnaire are reported in Tables 2 and 3. Of Hayle's respondents, 47 (18%) were aware of the show and 44 (17%) have listened to the show. Of those who have listened, 32 (73%) have listened more than once. Listeners rated the show's value with a modal score of 5 and a median score of 4 (out of 5). Of Newquay's respondents, 80 (13%) were aware of the show and 55 (8.7%) have listened to the show. Of those who have listened, 43 (78%) have listened more than once. The modal score for the perceived value of the show was 4 and the median score was 4. A higher proportion of sampled listeners are Hayle respondents (17%) than Newquay respondents (8.7%) which is statistically significant (*P* = 0.00063). Overall, of the 894 respondents, 127 (14%, 95% CI 11.9–16.5) are aware of the show and 99 (11%; 95% CI 9.0–13.1) have listened, the modal score for value was 4 and the median score was 4.

**Table 2:** The results of the study, in terms of the number of people aware of the show and the number of people listening in each of the two pharmacies

Pharmacy	Aware of mental health phone-in, *n* (%)	Of those aware, *n* (%) who have never listened	Of those aware, *n* (%) who have listened once	Of those aware, *n* (%) who have listened more than once
Hayle (*n* = 265)	47 (18)	3 (6.4)	12 (26)	32 (68)
Newquay (*n* = 629)	80 (13)	25 (31)	12 (15)	43 (54)
Overall (*n* = 894)	127 (14)	28 (22)	24 (19)	75 (59)

**Table 3:** The results of the study, in terms of the number of people listening to the show and the value that they place on it in each of the two pharmacies

Pharmacy	*n* (%) who have listened	Of those who have listened, *n* (%) that rate the value of the show as 1 on a scale 1–5^a^	Of those who have listened, *n* (%) that rate the value of the show as 2 on a scale 1–5^a^	Of those who have listened, *n* (%) that rate the value of the show as 3 on a scale 1–5^a^	Of those who have listened, *n* (%) that rate the value of the show as 4 on a scale 1–5^a^	Of those who have listened, *n* (%) that rate the value of the show as 5 on a scale 1–5^a^
Hayle (*n* = 265)	44 (17)	1 (2.3)	3 (6.8)	10 (23)	14 (32)	16 (36)
Newquay (*n* = 629)^b^	55 (8.7)	1 (1.9)	3 (5.6)	8 (15)	24 (44)	18 (33)
Overall (*n* = 894)^b^	99 (11)	2 (2.0)	6 (6.1)	18 (18)	38 (39)	34 (35)

a. The Likert scale being used is where 1 represents ‘not at all valuable’ and 5 represents ‘extremely valuable’.

b. Note that the total number of respondents does not sum to the total *n*, because one respondent did not give a score for how valuable they consider the show.

Table 4 compares those who have listened to the show with those that have not. There is no significant difference between the two populations in terms of gender, employment status or mental health status. The population that have listened to the show are significantly older (54.6 years *v.* 50.1 years; *P* = 0.01). Table 4 also compares those who have listened to the show with those who are aware of the show but have never listened. No significant difference was found between these two populations in any demographic (age, gender, employment status or mental health status).

**Table 4:** A comparison of the demographics between show listeners and those who do not listen and between show listeners and those who are aware of the show but have not ever listened^a^

Demographic	Those who have listened (*n* = 99)^a^	Those who have not listened (*n* = 795)^a^	Comparison between show listeners and those who have not listened	Those who have not listened but are aware of the show (*n* = 28)	Comparison between show listeners and those who have not listened but are aware
Age^b^ years, best estimate mean (s.d.)	54.6 (17.1)	50.1 (16.8)	0.01*	51.6 (19.0)	0.43
Gender, *n* (%)			0.39		0.41
Men	40 (41)	291 (36.6)		14 (50)	
Women	57 (59)	501 (63)		14 (50)	
Other	–	3 (0.38)		–	
Did not disclose	2	–		–	
Employment status, *n* (%)			0.35		0.85
Employed	45 (45)	399 (50)		12 (43)	
Unemployed	8 (8.1)	70 (8.7)		3 (11)	
Stay at home parent/carer	6 (6.1)	74 (9.2)		3 (11)	
Student	2 (2.0)	30 (3.7)		0	
Retired	38 (38)	229 (28.8)		10 (36)	
Did not disclose	1	3		–	
Mental health status			0.60		0.28
Have mental health difficulties in self/other	59 (60)	499 (63)		20 (71)	
Have no mental health difficulties in self/other	39 (40)	294 (37)		8 (29)	
Did not disclose	1	2			

a. Note that the total number of respondents do not necessarily sum to total *n*, either because some respondents did not answer this question or because, in the case of employment status, some respondents ticked more than one type.

b. The best estimate of the mean age assumes that the ages are evenly distributed in each class. For the >65 years age group, the class is assumed to be the age range 66–81 years, which uses the mean age of death based on Office of National Statistics data.^11^

*P < 0.05.

Each respondent completing the questionnaire was invited to give any additional comments as free text (Appendix 2). Of the listening Hayle respondents (*n* = 44), eight gave comments, all of which we perceived as positive. Of the non-listening Hayle respondents (*n* = 221), seven chose to give comments: three of these suggested that they would now listen to the show and three gave comments about the concept of the show that can be perceived as positive. Of the listening Newquay respondents (*n* = 55), ten chose to give comments of which nine can be perceived as positive. Of the non-listening Newquay respondents (*n* = 574), 43 chose to give comments. Of those 43 comments, 15 suggested that they would now listen to the show and 14 of these 15 (93%) have experience of mental health problems either personally or through family/friends. Similarly, 23 of the 43 respondents gave comments about the concept of the show that can be perceived as positive and 19 of these 23 (83%) have personal or family/friend experience of mental illness.

## Discussion

The aim of the BBC Cornwall mental health phone-in is to raise awareness of mental health and reduce stigma. The BBC has suggested that approximately 100 000 people are listening to the lunchtime show which, on a monthly basis, includes the mental health phone-in.^8^ This study has shown that 14% of those sampled are aware of the show and 11% have listened at least once. If this is representative of the population of Cornwall as a whole then this suggests that we are reaching approximately 70 000 people in the local population, of whom approximately 60 000 listen. However, some caveats and limitations of this study need to be considered.

### Limitations

The questionnaire completion rate (26%) is a limitation, particularly in Newquay (22%), introducing possible sampling bias into the study. It was not practical to collect data on pharmacy attendees who declined to complete the questionnaire and hence it is not possible to determine how representative the sample is of the pharmacy population as a whole. Furthermore, the pharmacy attendees in Hayle and Newquay may not necessarily be representative of the population of Cornwall. Those collecting prescriptions at a pharmacy are clearly those with health-related difficulties or at least those who are more willing to engage in health-seeking behaviour that may not be representative of the population as a whole. This could skew our data resulting in either an overestimate of the overall number of listeners (if non-medicated individuals in the general population tend to listen less than their medicated counterparts) or an underestimate (if non-medicated individuals tend to listen more). The former is probably more likely, although this cannot be determined from this study. The two pharmacies used were chosen pragmatically as the first two GP practices that accepted the request for assistance in the project. Although the demographics of the catchment populations of the two GP practices are broadly in-keeping with the demographics of Cornwall as a whole, the levels of deprivation in the catchment populations are somewhat lower: in Hayle, 8% of the population is in the highest fifth of deprivation in England, whereas 10% of Newquay and 15% of Cornwall as a whole satisfy this criteria.^14^

A further limitation is that there appears to be a significant difference in self-identified mental health status between the Hayle and Newquay respondents and this, therefore, raises the possibility of different associations in the two populations. Finally, the study may not be powered to detect differences for some of the comparisons made in the smaller sized groups (Table 4; *n* = 99 and *n* = 28).

It is worth mentioning that although smaller numbers attended in Hayle than anticipated (590 *v.* 1000), the response rate was higher than anticipated (45 *v.* 20%), hence the prerequisite minimum numbers of completed questionnaires was achieved (265 achieved; minimum 200). Newquay saw higher attendee numbers than anticipated (3390 *v.* 1000) and response rates as predicted (22% *v.* 20%), resulting in over three times the minimum number of completed questionnaires (629 achieved). Thus, despite the differences from predictions, the collected sample exceeded the requirements of the power calculations. However, the resulting sample may still not provide sufficient power to make comparisons when considering smaller subgroups.

### Interpretation of our findings

From responders who listen to the show, the feedback appears to be very encouraging with a median assigned value of 4, over 3 out of 4 (76%) of the listeners who had listened once listened again and nearly all (94%) of free-text comments being perceived as positive (Appendix 2).

Although no significant difference was found between respondents of the two practices with regards to age, gender and employment status, it is of interest to note that the reported level of mental illness is significantly higher in Newquay than Hayle respondents. This may relate to higher levels of deprivation reported in the Newquay area.^14^

Also of note, the younger age groups (18–35 years) consider themselves to have a significantly higher rate of mental illness than the older age groups (≥56 years). This could represent a true difference in rates of mental illness, it could reflect that the younger respondents are more willing to disclose mental illness or it could be that the younger respondents are more likely to consider themselves as having a mental illness. This finding is consistent with the findings of other major studies.^15^

The majority of the free-text comments from the questionnaires are very positive, as can be seen in Appendix 2. By far the most rewarding comment to read was one listener saying: ‘This show saved my life and marriage’. The most common themes to the positive comments were that the show was informative and that it increased awareness and understanding. It is interesting to note that a significant number of comments from non-listeners (18/50; 36%) were that they now intended to listen to the show, highlighting the importance of advertising. It is also important to note that several non-listening respondents suggested that the show timing is not wholly accessible, particularly for those in education or employment. Indeed, this may explain why the only demographic that was different between listening and non-listening populations is that the listeners are significantly older: this could be because older people as a whole are more likely to listen to BBC Radio Cornwall or could be because of the lack of accessibility of the lunchtime show to a younger population as they would be away at work or college.

### Implications

The BBC Radio Cornwall mental health phone-in programmes have continued over the past 8.5 years as a result of the perceived popularity from the public and positive anecdotal feedback. Assessing the impact of local media on subjects such as mental health in a scientific and evidenced-based manner is challenging and subject to bias and confounders. This is particularly true of mental health as it carries higher levels of stigma and prejudice, not only from the public but also from organisations. Nevertheless, despite limitations, this study has suggested that local radio projects such as this appear to be a successful way to reach a significant proportion of the population; they are likely to be well received and are effective in transcending communication barriers. This is a useful way to raise awareness and reduce stigma of mental health conditions in a cost-effective manner. The format could be easily adopted in various regions and could form an essential part of a public health strategy to improve a population's mental well-being. This has been proven by BBC Devon and the Devon NHS Partnership Trust now having adopted a similar format using the available business plan and learning. A similar initiative is now underway in Bristol.

With the addition of other trusts now following a similar model, this opens up the possibility for additional data collection and the possibility of a joint (and hence larger) study to assess what aspect of the shows do the listening population find particularly helpful. From the free-text comments in this study it can be speculated that the increased information, understanding and awareness is what listeners find particularly useful. However, this feedback is quite generic and in this study we have not specifically targeted listeners or asked listeners what they find most helpful. From a previous study^13^ in which callers into our programme were surveyed, some of the positive qualitative comments included that the programme was ‘useful’, ‘interesting’ and ‘helpful’; some of the negative qualitative comments were that the show was ‘too medical’ and that advice sounded ‘rehearsed’. Again, however, the listeners were not targeted with questions about what specifically was helpful and this feedback results merely from ‘free-text’ comments. One possible way to obtain such specific information would be to repeat our study here on a larger scale but only ask the pharmacy attenders to complete a questionnaire if they are a regular listener of the show. This questionnaire could then have specific questions to determine to what extent the show is helpful in a number of different aspects such as raising awareness, reducing stigma, being able to share with others, gaining information of resources, recognising symptoms, and such like and which of these aspects are important to them as a listener. An alternative approach would be to again target callers to the show, as previously,^13^ but with these specific questions. However, the disadvantage of this latter approach would be that it would be biased towards the small proportion of listeners who are also callers.

The results of this study have opened new avenues of thinking to further develop the project. First, the potential importance of advertising outside of BBC Radio Cornwall has been highlighted and this needs to be further explored. Second, although the programmes are available for 30 days after airing via the internet (BBC Sounds), alternative ways need to be explored to increase the accessibility of the programmes locally, nationally and internationally. This could include, for example, a website on which all previous shows on the various mental health topics are held permanently. Such a website could be advertised internationally and could contribute to mental health education across the globe.

## Appendix 1

### BBC Radio Cornwall Mental Health Phone-In Questionnaire

Thank you for helping Cornwall Partnership NHS Foundation Trust (CFT) assess your opinion about the BBC Radio Cornwall Mental Health Phone-in which has been on air every 1st Wednesday of the month on the Laurence Reed Show since 2011. It does not matter if you are a listener or not. We would like to know your thoughts and a little about you. This questionnaire is completely anonymous and will be used for an evaluation project on mental health and the media run by CFT. More detail on this can be found on http://www.cornwallft.nhs.uk/wellbeine/vour-healthyradio-cornwall-mental-health-phone/

Please circle the response closest to your views.

**Q1. What age group are you in?**

26–35   36–45   46–55   56–65   Over 65

**Q2. How do you describe your gender?**

Male    Female    Other: _______________

**Q3. What is your employment status (please circle all that apply)?**

Employed Unemployed Stay at home parent/carer Student Retired

**Q4. Have you, a friend or relative been affected by mental health difficulties? (please circle all that apply)**

No Yes –1 have been affected Yes – a friend or relative has been affected

**Q5. Are you aware of a monthly mental health phone-in programme on BBC Radio Cornwall?**

Yes    No

**Q6. How many times, if ever, have you listened to this programme?**

Never  Once  Twice or more

**Q7. If you have listened, on a scale of 1–5 (1 least, 5 most) how valuable do you find this show?**

1 (not at all valuable) 2 3 4 5 (extremely valuable)

**Q8. Any other comments about the show or the concept?**

Thank you for taking the time to complete this questionnaire.

## Appendix 2

### Comments given by respondents as free text in the questionnaire

**Table:**

Pharmacy	Demographic of commenter	Comment made
Hayle	Listener	It's very good for information – to ask questions.
Hayle	Listener	The show saved my life and marriage.
Hayle	Listener	It feels better not to feel alone, that others feel the same, that the show is informative/empathic.
Hayle	Listener	My sister says it's good.
Hayle	Listener	Great to see how mental health is being made aware of these days.
Hayle	Listener	Helps with understanding of mental health issues. Feel so sorry for some people – very sad.
Hayle	Listener	Always very carefully covered. Lots of phone numbers given out. Very valuable for the people of Cornwall.
Hayle	Listener	Fantastic show.
Hayle	Non-listener	Keep doing a good job.
Hayle	Non-listener	Very enjoyable show and covers every aspect of people's concerns.
Hayle	Non-listener	Not my taste.
Hayle	Non-listener	Didn't know about it, work in mental health and will listen and if beneficial will recommend.
Hayle	Non-listener	Mental health in our young children (8–11) is a growing problem also.
Hayle	Non-listener	The concept is brilliant.
Hayle	Non-listener	Great that this type of health problem is getting more attention.
Newquay	Listener	What training has Laurence received in mental health counselling? He patronises callers but could do more harm than good.
Newquay	Listener	Seems a good idea.
Newquay	Listener	Great idea to bring mental health issues to the public. Nice to hear others speak about it.
Newquay	Listener	Useful and informative programme.
Newquay	Listener	Very good for people.
Newquay	Listener	Keep it going.
Newquay	Listener	Excellent.
Newquay	Listener	Great.
Newquay	Listener	An excellent idea.
Newquay	Listener	Very helpful.
Newquay	Non-listener	Good concept probably.
Newquay	Non-listener	Not 12.30 PM.
Newquay	Non-listener	Now I know about the MH [mental health] programme I will endeavour to listen to the show.
Newquay	Non-listener	Good idea but needs to be advertised more.
Newquay	Non-listener	Will listen next time.
Newquay	Non-listener	Although I have not listened to this show, I think these programmes are valuable.
Newquay	Non-listener	I have heard them on other stations, sometimes get hijacked by very unwell people or people who have PD [personality disorder] diagnosis.
Newquay	Listener	A very good idea.
Newquay	Listener	Keep up the good work.
Newquay	Non-listener	Good for raising awareness, needs to be more advertised?
Newquay	Listener	I support concept (and show).
Newquay	Non-listener	It sounds like a good idea.
Newquay	Non-listener	Good idea - for this type of programme.
Newquay	Non-listener	Will tune in and listen.
Newquay	Non-listener	Keep up the good work, more awareness needed.
Newquay	Non-listener	I'm going to tune in now though.
Newquay	Non-listener	Seems to be a good idea and should be more widely advertised.
Newquay	Non-listener	This is awesome and I now shall listen.
Newquay	Non-listener	Good idea, limited audience.
Newquay	Non-listener	Maybe consider at different times of the day too for workers.
Newquay	Non-listener	Will be listening.
Newquay	Non-listener	Sounds great.
Newquay	Non-listener	Great idea, I will listen in.
Newquay	Non-listener	Good concept, not wholly accessible for younger age group.
Newquay	Non-listener	No, except I would have thought it can only be helpful.
Newquay	Non-listener	Shame not to have heard about it. More publicity.
Newquay	Non-listener	Much needed.
Newquay	Non-listener	Will listen now.
Newquay	Non-listener	Great idea.
Newquay	Non-listener	Never listened but will in future.
Newquay	Non-listener	Will start to listen ASAP.
Newquay	Non-listener	Anything that raises awareness of mental health is a good thing.
Newquay	Non-listener	Maybe more awareness needs to be made of this programme.
Newquay	Non-listener	Will find out and listen.
Newquay	Non-listener	Great idea.
Newquay	Non-listener	Interested. Brilliant idea.
Newquay	Non-listener	Maybe more awareness of this service and what they inform.
Newquay	Non-listener	I shall listen to the programme and perhaps tell my friend (Q4).
Newquay	Non-listener	I will do now knowing it is available. Many thanks.
Newquay	Non-listener	I think this is a brilliant idea. More awareness is always good.
Newquay	Non-listener	If it is public people won't want to phone in because problems are personal.
Newquay	Non-listener	Never heard about it but I will look into it now that I know.
Newquay	Non-listener	I will tune in this week to listen.
